# NICO Myriad Automated Preservation System improves meningioma sample quality for molecular pathology assays

**DOI:** 10.1093/jnen/nlag030

**Published:** 2026-07-20

**Authors:** Glaucia Maria de Mendonça Fernandes, Kyle C Wu, Joshua Palmer, Ricardo Carrau, Maria Clara Franco, Maria M Abreu, Michael W McDermott, Michele Joana Alves, José Javier Otero, Daniel M Prevedello

**Affiliations:** Department of Cellular and Molecular Medicine, Florida International University Herbert Wertheim College of Medicine, Miami, FL, United States; Department of Neurological Surgery, The Ohio State University Wexner Medical Center and James Cancer Center, Columbus, OH, United States; Department of Radiation Oncology, The Ohio State University Wexner Medical Center and James Cancer Center, Columbus, OH, United States; Department of Otolaryngology, Head and Neck Surgery, The Ohio State University Wexner Medical Center and James Cancer Center, Columbus, OH, United States; Department of Cellular and Molecular Medicine, Florida International University Herbert Wertheim College of Medicine, Miami, FL, United States; Center for Translational Science, Florida International University, Miami, FL, United States; Miami Cancer Institute, Baptist Health South Florida, Miami, FL, United States; Baptist Health Medical Group, Baptist Health Quality Network at Baptist Health South Florida, Miami, FL, United States; Department of Neuroscience, Florida International University Herbert Wertheim College of Medicine, Miami, FL, United States; Department of Cellular and Molecular Medicine, Florida International University Herbert Wertheim College of Medicine, Miami, FL, United States; Department of Neuroscience, Florida International University Herbert Wertheim College of Medicine, Miami, FL, United States; Department of Neuropathology and Clinical Informatics, Baptist Health South Florida, Miami, FL, United States; Department of Neurological Surgery, The Ohio State University Wexner Medical Center and James Cancer Center, Columbus, OH, United States

**Keywords:** Automated Preservation System, circular RNA, epitranscriptome, fresh-frozen tissue, meningioma, NICO Myriad

## Abstract

Meningiomas are central nervous system tumors primarily treated by surgery and/or radiation with large variance in progression-free survival. Emerging molecular diagnostics such as circular RNA (circRNA) profiling show potential for enhancing diagnostic accuracy but are limited by the processing workflows associated with traditional fresh-frozen protocols that may lead to biomarker degradation. The NICO Automated Preservation System (APS) is a surgical tool that aspirates and resects intact tumor cores, quickly storing the removed tissue in a refrigerated environment of Ringer’s Lactate fluid. To overcome the challenge of obtaining and preserving tissue for molecular diagnostics, we sought to test the extent to which the NICO APS enhances tissue preservation versus traditional protocols in 20 meningioma resections; we focused on circRNA profiles as potential clinical biomarkers. The NICO APS enhanced RNA integrity (*P* = .0023), yielding high-quality data (>Q30 median = 95%), which reduced the variation of circRNA detection. Weighted Gene Co-expression Network Analysis identified a total of 4 modules highlighting NICO and traditional protocols and meningioma grades. Modules belonging to WHO grade 2 meningiomas may be associated with cellular metabolism and epitranscriptomic regulation. Our results show that circRNA detection is highly improved using NICO APS.

## Introduction

Meningioma are primary brain tumors, traditionally classified between the grades 1 and 3 using mitotic index and cytoarchitectural features according to the fifth edition of the Central Nervous System World Health Organization (WHO) Classification of tumors.^1–5^ This grading system has been fraught with caveats that have hindered pathologists’ ability to guide clinical decision-making, ultimately impacting patient outcomes.^6^^,^^7^ To further refine prognostic predictions, a multimodal, integrated biomarker workup that includes chromosomal structural variant detection, gene expression analysis, and whole genome methylation assays, coupled with machine learning classifications algorithms has been recommended.^1–3^^,^^7–9^ Through these analyses, distinct subgroups that correlate with patient outcomes have been identified^10^; many of these have been incorporated in a recent cIMPACT-NOW neuropathology practice guideline recommendation.^2^ Although a significant improvement, patients show variable outcomes. For instance, analyzing the progression-free survival data provided in Landry et al., who studied prior to segregating patients based on 1p and 1q status, 421 of 1157 (∼36.3%) of grade 1 meningioma patients showed progression after 2 years when compared with 264 of 556 patients (∼44%) for grade 2 and 99 of 163 patients (∼60%) for grade 3.^7^ Integrating morphology and genomics with machine learning-based diagnostic algorithms has improved prognostication and refinement of therapeutic strategies in many cancer paradigms.^11^^,^^12^ Thus, additional biomarkers are needed to improve clinical decision-making.

Circular RNAs (circRNAs) have important and complex regulatory roles in cancer and the tumor microenvironment.^13–16^ Brain tissue shows enrichment for circRNA transcripts.^17^^,^^18^ CircRNAs modulate cell function by sponging microRNAs, interacting with RNA-binding proteins and regulating pathways such as PI3K/AKT, Wnt/β-catenin, and MAPK, thereby supporting brain development, differentiation, synaptic plasticity, and neuroinflammatory homeostasis.^19–22^ In brain tumors, their dysregulation promotes proliferation, invasion, and apoptosis resistance, underscoring their dual roles as regulators of cell physiology and potential biomarkers or therapeutic targets in gliomas.^19^^,^^22–24^ The circular nature of circRNA is formed through a back-splicing junction (BSJ) modification, a feature that enables protection from exonuclease-based degradation ultimately leading to their increased stability.^25–28^ Typical circRNA detection assays involve incubating bulk RNA containing linear and circRNA transcripts with RNA exonucleases incapable of degrading circRNA variants.^29^ Most clinical RNAseq assays perform mRNA enrichment through poly-A tail captures strategies to improve accuracy and detection.^30^ Poly-A tail enrichment unfortunately precludes circRNA from analysis. Relative to linear RNA, circRNA shows increased stability, making this class of RNA molecules potentially optimal disease biomarkers.^31^

The detection and large-scale quantification of circRNAs are often based on short-read RNA sequencing data which requires fresh-frozen tissue. However, the incorporation of fresh-frozen tissue collection workflows into routine pathology processes present significant logistical challenges. In contrast, standard pathology workflows using formalin-fixed, paraffin-embedded (FFPE) processing introduce fixation-related artifacts that compromise and limit the reliability of circRNAs detection. The use of fresh-frozen tissue is a gold standard for RNA-based molecular profiling and is required for RNAseq assays of circRNA.^14^^,^^22^^,^^32^^,^^33^ Importantly, pre-analytical factors during surgical procurement, including tissue handling, temperature, and time prior to stabilization, can substantially affect RNA integrity.^34–36^ Recent innovations in neurosurgical devices could improve technical and logistic challenges. The NICO Myriad with Automated Preservation System (APS) is a device used for tumor resection that can collect tissue directly into chilled buffers. These features raise the possibility that the NICO APS could improve tissue preservation, consequently increasing the consistency and fidelity of molecular assays. The present study evaluates the changes in circRNA quality in fresh tissue collection using traditional and NICO APS-based methodologies during surgical resections of meningiomas. We assessed RNA integrity number (RIN) and RNA length to determine whether the NICO APS improves RNA preservation and maintains transcript integrity. We also analyzed circRNA expression patterns across the combined cohort to explore circRNA profiles in meningiomas.

## Methods

### Sample collection

The NICO APS using the Myriad system is an advanced, minimally invasive tumor resection device with a 0.6-mm cutting and aspiration aperture near the blunt end; this enables tissue removal while preserving adjacent critical structures and maintaining tissue integrity and molecular contents. Meningioma tissue samples were collected from 20 patients undergoing surgery at The Ohio State University following approval from the University’s Institutional Review Board (IRB 2020C0150). Two samples were obtained from each individual patient: one using the NICO APS system (Stryker, Kalamazoo, MI) and one using traditional procurement methods (Figure 1). The traditional protocol for tissue procurement of tumor resection includes the surgical team placing the tissue onto a Telfa nonstick pad or gauze at room temperature prior to laboratory freezing. No additional procedures were introduced to standardize ischemia times, as tissue handling followed routine standard operating procedures (in this case flash freezing in liquid nitrogen after transport at room temperature to the biorepository). In parallel, specimens extracted by the NICO APS device were stored in an ice-chilled Ringer’s lactate solution, transported to the biorepository, and frozen. These procedures were separate from the resection sent to anatomic pathology for neuropathological interpretation. This material was then diagnosed using morphological criteria delineated in the 2021 WHO classification scheme (Table 1).

**Figure 1:**
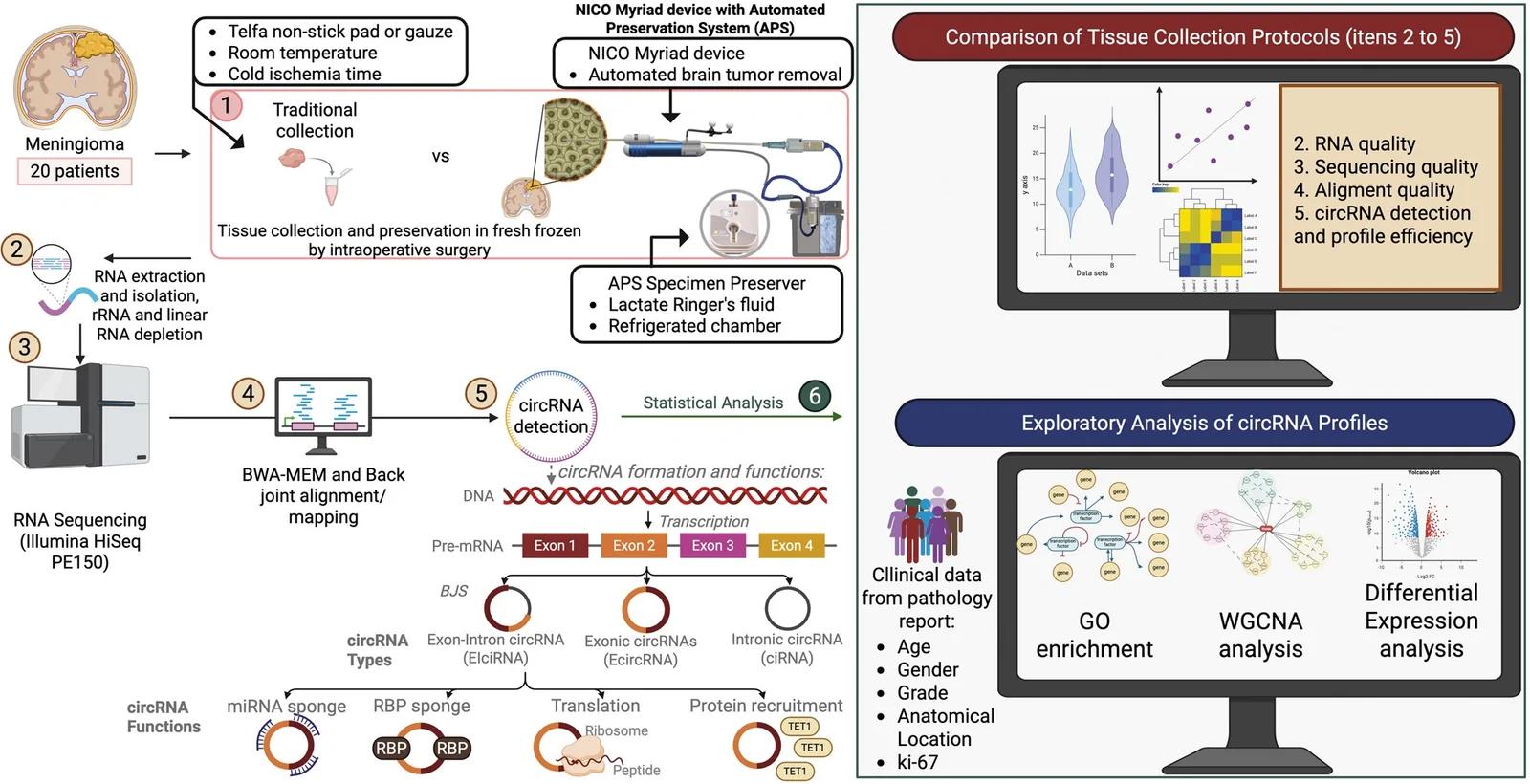
Workflow for comparative analysis between Traditional and NICO APS protocols. To compare the impact of tissue preservation on RNA extraction quality and circRNA profiling, we collected 20 meningioma samples using 2 distinct protocols: the Traditional Surgical Protocol and the NICO APS (Equipment image sourced from the NICO Corporation webpage: https://niconeuro.com/our-integrated-system/). In both cases, tissue was excised during surgery. For the traditional method, tissue was manually dissected and placed onto a Telfa nonstick pad or gauze at room temperature prior to laboratory freezing. For the NICO APS method, tissue was collected using the Myriad device, which enables automated tissue collection and preservation with minimal manipulation, enhancing RNA integrity. All samples were stored in cryovials and banked at −80°C until RNA extraction. RNA was extracted using standardized protocols ensuring high integrity (RIN > 7), followed by circRNA library preparation and sequencing. To minimize batch effects, all samples underwent normalization and batch correction prior to downstream analysis. Co-expression networks were constructed using WGCNA, and modules significantly associated with clinical features were subjected to GO and KEGG enrichment analyses. Created in https://BioRender.com.

**Table 1:** Clinical information from the meningioma patients.

Case	Samples ID	Age	Gender	ki-67	WHO grade	Anatomical Brain location
**Case 1**	FF07788695	50	Female	NI	1	Skull base
**Case 2**	FF07788835	48	Male	2%	1	Skull base
**Case 3**	FF07172031	61	Male	1%	1	Skull base
**Case 4**	FF07172261	50	Female	1%	1	Skull base
**Case 5**	FF07789077	46	Male	NI	2	Skull base
**Case 6**	FF07788864	74	Female	5%-7%	1	Skull base
**Case 7**	FF07171839	50	Female	1%	1	Skull base
**Case 8**	FF07171890	40	Female	1%-2%	1	Skull base
**Case 9**	FF07172300	65	Male	8%	1	Frontal and parietal lobe
**Case 10**	FF07794057	41	Male	3%-4%	1	Frontal lobe
**Case 11**	FF07794015	68	Female	4%	1	Skull base
**Case 12**	FF07794427	67	Female	8%	2	Frontal lobe
**Case 13**	FF07794214	53	Male	8%	1	Skull base
**Case 14**	FF07793989	63	Female	8%	1	Frontal and temporal lobe
**Case 15**	FF07794443	80	Male	1%	1	Skull base
**Case 16**	FF07794260	39	Female	3%	1	Skull base
**Case 17**	FF07794056	78	Female	3%-4%	2	Frontal lobe
**Case 18**	FF07794023	48	Female	6%	1	Frontal lobe
**Case 19**	FS64490124	57	Female	5%-6%	1	Skull base
**Case 20**	FF07789016	45	Female	3%	1	Frontal lobe

Abbreviation: NI, no information.

### RNA extraction and sequencing

#### RNA extraction and quality

RNA extraction and quality control were performed at CD Genomics. Briefly, tumor tissue was thawed and total RNA was isolated from each sample using routine protocols. To minimize RNA degradation, all procedures were performed on ice, using RNase-free reagents. RNA concentration and purity were measured by NanoDrop (Thermo Fisher Scientific) and RIN testing on an Agilent 4200 (Agilent Technologies). Only samples with total amount ≥ 2.0 μg, RIN ≥ 6.5, and 28S:18S ≥ 1.0 was considered in high quality to continue the libraries preparation and proceed to sequencing. We compared the quality of RNA samples between the protocols, evaluating 4 key metrics: (1) total RNA yield (µg), (2) RNA concentration (ng/µL), (3) RIN, and (4) the absorbance ratio at 260/280 nm. Additionally, we conducted linear regression analyses to investigate the correlation between the protocols for each metric, and the goodness of fit was evaluated through the coefficient of determination (*R*^2^) and *P* values.

#### RNA sequencing and quality

Aliquots of total RNA from each sample were digested with RNase R to selectively eliminate linear RNAs, thereby enriching for circRNAs, and subsequently subjected to high-throughput RNA sequencing using the Illumina HiSeq PE150 platform (Illumina, Inc.) by CDGenomics. Sequencing libraries were prepared, and the raw reads obtained were processed through quality control filters to remove low-quality sequences, adapters, and contaminants. Sequence alignment was performed using the CIRI2 pipeline, a bioinformatics tool specifically designed to identify circRNAs.^37^ CIRI2 detects back-spliced junction (BSJ) reads, which represent the hallmark of circRNAs. In these reads, the 3′ splice site of a downstream exon is covalently joined to the 5′ splice site of an upstream exon, producing a non-linear junction that cannot occur in canonical linear splicing. This BSJ signature is recognized by CIRI2 through sequence alignment performed with the BWA-MEM algorithm, a widely used short-read aligner.^38^

The following metrics were computed for each sample to assess the quality of the sequencing data as commonly reported in RNA-seq quality-control pipelines.^39–43^ Clean Reads indicate how well the RNA was preserved during extraction and how many sequences can be used in downstream bioinformatics analysis. Clean Base represent both the length and quality of the clean reads.^42^^,^^43^ The Phred quality score ≥30% (≥Q30) indicates the percentage of bases with a sequencing error probability ≤0.1%, providing a measure of sequencing accuracy.^41^ GC Content, a known modulator read accuracy and coverage, reflects the percentage of guanine and cytosine bases.^39^^,^^40^ We also conducted a comparative analysis of circRNA quantification detection between the 2 procurement protocols. Furthermore, we performed a Pearson correlation analysis to assess the correlation between the circRNA counts obtained from both methods, as shown in the dispersion plot with linear model curve. Lastly, we categorized the detected circRNAs based on their genomic origin (exonic, intronic, or intergenic) for each method.

### Normalization and batch effects removal

RNA extraction and circRNA sequencing were performed in 2 separate batches for NICO APS or Traditional protocol. To further eliminate technical variations caused by batch effects of the biopsy extraction protocol (NICO APS or Traditional), circRNA expression data were normalized using quantile normalization following batch effect removal using the ComBat method from the *sva* package in R. This method minimizes nonbiological systematic variations between the samples while preserving the biological differences of different experimental conditions.^44^^,^^45^ The effectiveness of both normalization and batch correction was evaluated using boxplots, relative log expression (RLE), and comparative principal component analysis (PCA) plot.

### circRNA expression

#### circRNA expression profile

We performed a hierarchical cluster analysis to compare the circRNA expression profiles between the protocols and visualized the clustering with heatmap using the *pheatmap* R package. To assess clustering structure, we applied PCA and quantified intra-cluster compactness using the mean Euclidean distance from each point to its respective cluster centroid, while inter-cluster separation was evaluated by calculating the Euclidean distances between cluster centroids. We also performed the silhouette width analysis to evaluate the quality of clustering, measuring how well each sample fits within its assigned group. Values close to 1 indicate well-clustered samples, while negative values suggest potential misclassification.

#### circRNA differentially expressed

For the differential circRNA expression, we compared the NICO APS versus Traditional protocol, and the WHO grade 1 versus grade 2 using the *DESeq* package and visualized using the *EnhancedVolcano* package.

#### circRNA co-expressed network

Weighted Gene Co-expression Network Analysis (WGCNA) was performed to identify modules of highly correlated circRNAs and explore their associations with clinical traits using the *WGCNA* package in R Studio. The analysis involved constructing a co-expression network, where pairwise correlations between circRNAs were computed using Pearson’s correlation. Hierarchical clustering was used to group circRNAs with similar expression profiles into distinct modules, represented by different colors. For each identified module, module eigengenes were calculated as the first principal component of the module’s expression profile.^46^ Pearson correlation analysis was then employed to associate module eigengenes with clinical characteristics.^46^^,^^47^ The statistical significance of these correlations was further validated using ANOVA and *t*-test to confirm differences between protocols in each significant module color for each clinical trait. Additionally, functional enrichment analyses, including Gene Ontology (GO) and Kyoto Encyclopedia of Genes and Genomes (KEGG) pathway analysis, were performed to investigate the biological roles of circRNAs within the significant modules. These steps provided insights into the potential involvement of circRNA modules in key biological pathways and their associations with clinical outcomes.

### Statistical analysis

For the statistical analysis, data from the 2 extraction protocols (NICO APS and Traditional) were grouped and analyzed across the key metrics associated with RNA extraction, sequencing, and circRNAs identification quantity and quality. Violin and box plots were used to visualize the distribution using 2-sample *t*-test to determine if there were significant differences between the groups. Scatterplots show the relationships in linear regression lines fitted, R-squared (*R*^2^) or the Pearson correlation (*R*), and *P*-values included to assess correlation strength and statistical significance. The R programming environment was used for all data analysis, utilizing packages such as *ggplot2* for visualization, *dplyr* for data manipulation, *stats* for t-tests, and *broom* for regression analysis.

## Results

### NICO APS protocol generates better RNA, sequencing, and detection quality

To examine whether the biopsy procurement from NICO APS protocol could improve the RNA integrity and quality, we compared RNA quality metrics from our tissue extractions (Figure 2). Similar RNA quantity was identified from the 2 protocols (Figure 2A and B). The NICO APS protocol yielded significantly higher mean RIN values compared to the Traditional protocol (*P* = .0023, Figure 2C), indicating superior RNA integrity. The 260/280 absorbance ratio, a measure of RNA purity, did not show difference between the methods (*P* = .14, Figure 2D). Furthermore, when comparing these metrics in the samples from the same patient but processed differently, very little correlation between process workflow is noted (*P* > .05 in correlation plots Figure 2A-D).

**Figure 2:**
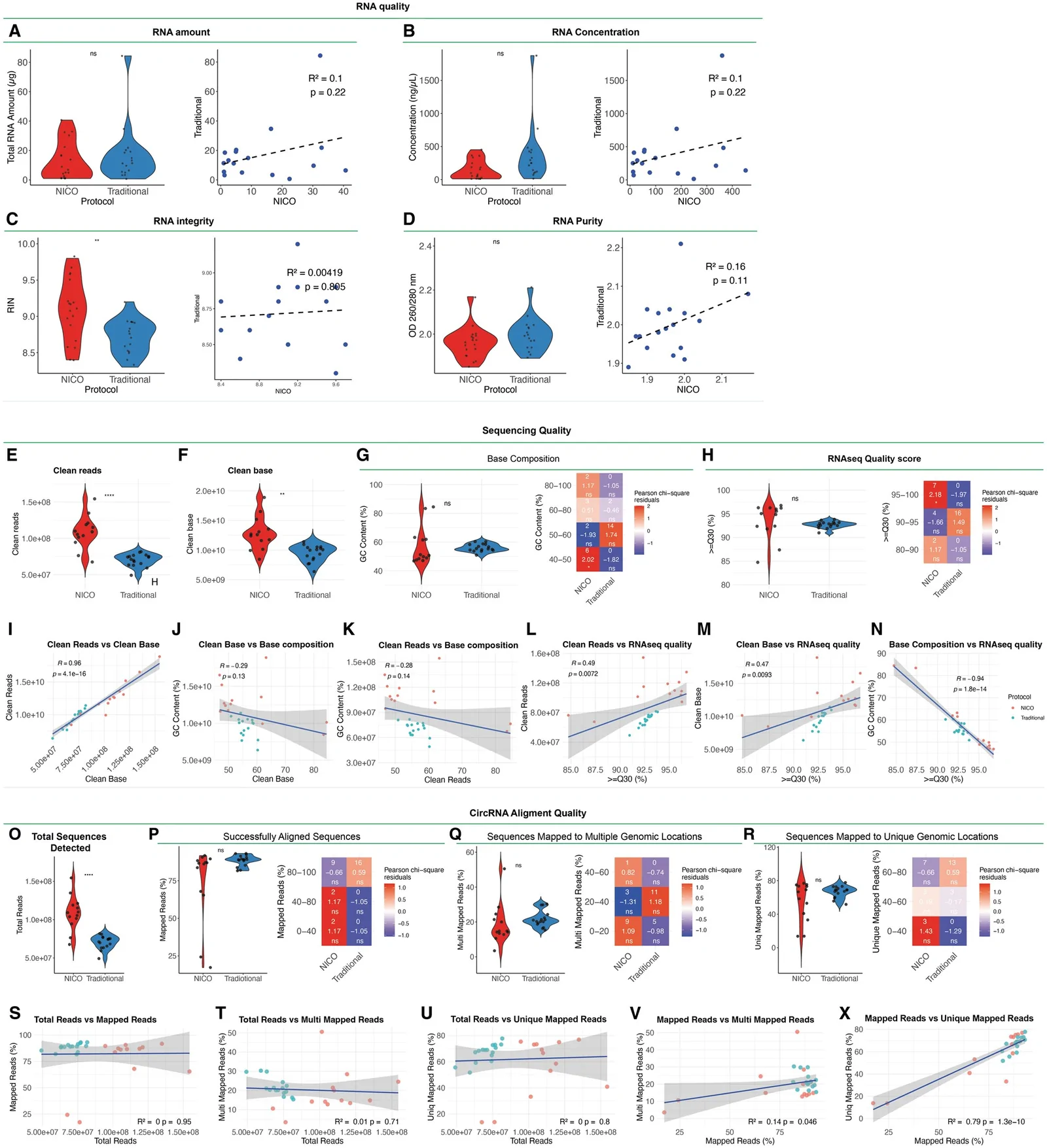
RNA quality analysis: Violin and scatter plot comparing the Traditional (*n* = 20) and NICO APS (*n* = 20) protocols related to (A) RNA amount (µg), (B) RNA concentration (ng/µL), (C) RNA integrity, and (D) 260/280 (nm) absorbance rate. RNA sequencing quality: Violin plot and/or heatmap comparing the Traditional (*n* = 15) and NICO APS (*n* = 13) protocols related to (E) Clean Base, (F) Clean Base, (G) GC content, and (H) ≤Q30. The scatter plots between Traditional and NICO APS protocol present the linear regression results to correlated (I) Clean Reads and Clean Base, (J) Clean Base and GC Content, (K) Clean Reads and GC Content, (L) Clean Reads and ≥Q30, (M) Clean Base and ≥Q30, and (N) GC Content and ≥Q30. circRNA alignment quality: Violin plot and/or heatmap comparing the Traditional and NICO APS protocol related to (O) Total reads, (P) Mapped Reads, (Q) Multi Mapped Reads, and (R) Unique Mapped Reads. (S) Total reads vs Mapped Reads, (T) Total reads vs Multi Mapped Reads, (U) Total reads vs Unique Mapped Reads, (V) Mapped Reads vs Multi Mapped Reads, and (X) Mapped Reads vs Unique Mapped Reads. *T*-test were used for specific groupings and significant effects are indicated by ns = nonsignificant; *P < .05; **P < .01; ***P < .001; **** *P* < .0001.

### NICO APS results in superior sequencing quality

To compare the sequencing quality between Traditional and NICO APS protocols, we analyzed Clean Reads, Clean Bases, GC content, and Q30 values using *t*-test. We also visualize the data in violin plots, and Pearson χ^2^ residuals with distributional enrichment heatmap across percentage intervals. The violin plots illustrate the NICO APS protocol generated significantly more Clean Reads and Clean Bases than Traditional protocol as illustrated in Figure 2F (*P* < .001, Figure 2E and F). No significant difference was noted in mean GC content and mean ≥ Q30 score (*P* > .05, Figure 2G and H). However, the Pearson correlation χ^2^ residuals heatmaps indicated differences in distributional enrichment across GC content and ≥Q30 score. NICO APS samples were enriched at lower GC content and higher ≥Q30 intervals (*P* < .01, Figure 2G and H).

The Pearson correlation revealed a strong positive association between Clean Reads and Clean Bases by the samples derived from the NICO APS protocol (*R* = 0.96, *P* < .0001, Figure 2I). This relationship between Clean Reads and Clean Bases indicates improved sequencing consistency and yield. The number of Clean Reads and Clean Base did not correlate with GC Content for either protocol (*R* = −0.28 and −0.29; *P* > .1, Figure 2J and K). For circRNA analysis, where accurate base calling is required to detect unique circular junctions, high Q30 scores are particularly beneficial to minimize sequencing errors. When binned based on quality score, the majority of the NICO APS-derived samples showed RNAseq quality (Q30 score) that were higher and correlated with the high Clean Reads and Clean Bases (Figure 2L and M). A strong negative correlation was also observed between the GC content and Q30 (*R* = −0.94, *P* = 1.8e−14, Figure 2N). These findings are consistent with established RNA-seq patterns, where lower GC content generally corresponds to higher base-calling accuracy and Q30 values.^39^ These results indicate that enhanced Clean Reads and Bases in the NICO APS protocol contribute to higher Q30 values, improving the reliability of RNA detection and circRNA profiling.

### NICO APS protocol improves sensitivity of circRNA detection

In circRNA detection, quality RNA fragments are essential for accurate identification and quantification. To this end, we first compared Traditional and NICO APS protocols across key alignment metrics by evaluating these differences using 2-sample *t*-tests. The violin plot illustrates NICO APS protocol produced significantly more total reads than the Traditional protocol (*P* < .0001, Figure 2O). This greater “read yield” is consistent with improved RNA recovery and library preparation efficiency in the NICO APS protocol relative to the Traditional protocol. Greater yield is an important advantage for circRNA profiling, where transcripts are typically less abundant. Multi‑mapped percentage and unique-mapped percentage showed no significant differences between the 2 protocols (violin plot, *P* > .05, Figure 2P and Q). Overall, these findings suggest that the differences between the Traditional and NICO APS protocols are primarily driven by sequencing yield rather than by systematic changes in alignment performance.

These results raised the question of whether the increased read out observed with NICO APS confer advantages relevant to circRNA analysis. After establishing that overall alignment metrics were comparable between protocols, we examined how these metrics relate to one another across samples and whether their interrelationships differ by procurement protocol. We addressed this question by applying linear regression models, visualized with scatter plots with 95% confidence intervals (CIs), to evaluate the associations among alignment metrics. Weak or no association were observed for the raw total reads and mapped reads percentage (*R*^2^ = 0; *P* = .95, Figure 2S), multi-mapped reads percentage (*R*^2^ = 0.01; *P* = .71, Figure 2T) or uniquely mapped reads percentage (*R*^2^ = 0, *P* = .8, Figure 2U). The relationship between mapped-reads percentage and multi-mapped percentage was weak (*R*^2^ = 0.14; *P* = .05, Figure 2V), whereas a strong and significant association was found between mapped-reads percentage and uniquely mapped percentage (*R*^2^ = 0.79; *P* = .001, Figure 2X). We conclude that the interrelationships between alignment metrics does not differ by procurement protocol.

Because total reads count has little to no explanatory power for alignment quality metrics, these findings suggest that mapping efficiency and specificity are depend mainly on library quality and alignment parameters, rather than sequencing raw data alone.^48^ Overall, and consistent with RNAseq quality metrics, NICO APS protocol demonstrates yield‑driven sensitivity with lower multi-mapping, whereas Traditional protocol provides greater consistency across mapping percentages (Figure 2L and M).

### NICO APS protocol enables high accuracy in circRNA detection

To quantify the circRNAs aligned to the genome, we compared alignment metrics, including the number and diversity of circRNAs detected using CIRI2. Differences between conditions were then assessed using 2-sample *t*-tests and Pearson correlation. NICO APS protocol demonstrated a smaller number of circRNAs, but with less variance (Figure 3A and B), indicating a more consistent distribution of detected circRNAs. Although can overlapping of circRNA was detected across the protocols (*R* = 0.47, *P* < 2.2e−16) and an enhanced of exonic regions, NICO APS protocol presented higher mean of circRNA expression (Figure 3C) and higher frequency of exonic regions (Figure 3D) than the Traditional protocol. These findings indicate that NICO APS protocol offers higher confidence and/or potentially more true-positive detections, while the traditional technique shows a wider variance in the number of detected circRNAs. The Traditional technique also exhibits significant variability between samples, suggesting it may be less consistent or more influenced by sample-specific factors. However, it may also reflect greater biologically relevant diversity in circRNA species.

**Figure 3:**
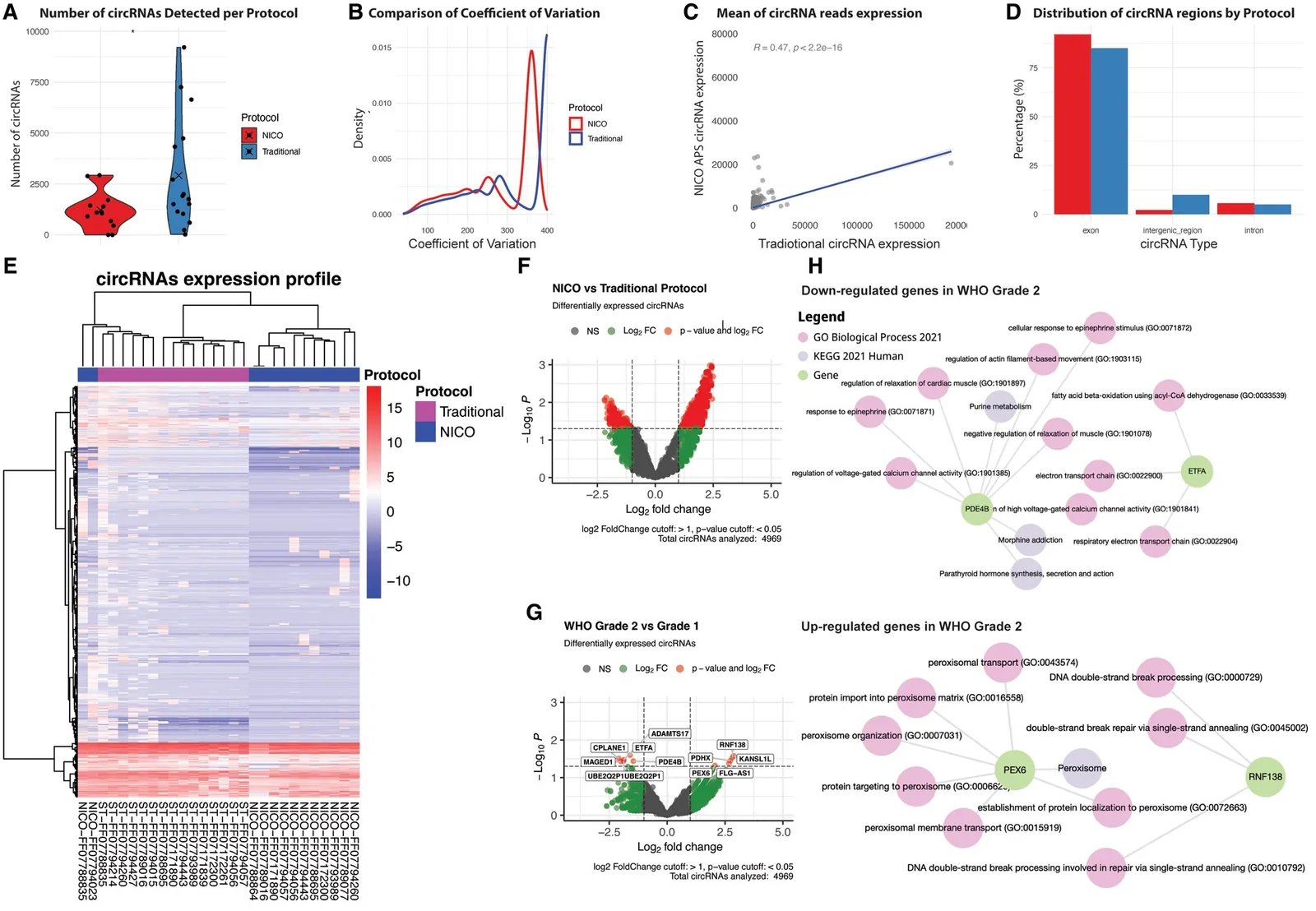
circRNA analysis. (A) number of circRNAs detected (NICO APS = 6293, Traditional 17168), (B) diversity of circRNAs, and (C) the scatter diagram showing the circRNA correlation coefficient between samples. (D) Bar plot showing the most distribution of the circRNA by type of region. (E) Heatmap showing the circRNA expression profile in meningioma (*n* = 4969 circRNAs). Volcano plot showing the differential circRNAs expressed between (F) Protocol and (G) WHO grade meningioma. (H) Network graph showing the GO and KEGG pathway enriched up or down expressed genes in the WHO grade analysis.

To further comprehend the differential expression between the protocols, we evaluated batch effects introduced by the NICO APS and Traditional surgical protocols. We applied the ComBat method that allows data harmonization by removing site-specific variance and we performed quantile normalization (Figure S1). The combination of ComBat and quantile normalization effectively harmonized the datasets, enabling more reliable circRNA comparisons between the 2 biopsy methods.

We next sought to determine whether the NICO APS protocol improved the consistency and reproducibility of circRNA expression compared to the Traditional protocol. To test this, we performed hierarchical cluster analyses evaluating both protocols together after batch effect mitigation (Figure 3E). The results show that the NICO APS protocol demonstrated superior performance in generating consistent and reproducible circRNAs expression. We then evaluated technical noise through PCA. Figure S2A illustrates the PCA of inter- and intra-cluster analyses. We calculated the mean cluster Euclidian distance, which revealed 3 clusters: clusters 1 and 3, consisting exclusively of Traditional and NICO samples, respectively, were relatively compact compared to the cluster 2 that had more Traditional samples clustered. Additionally, cluster 3, exclusively with NICO samples, was more distinctly separated from the other clusters. We also calculated the silhouette width by cluster, which is a measure of cluster compactness.^49^ These data are shown in Figure S2B. These findings suggest less technical noise in NICO APS protocol compared to the Traditional protocol, indicating that the NICO APS protocol provided a superior analytical circRNAs analysis in tumor biopsies.

We next analyzed the circRNA profiles and differential expression for both Traditional and NICO APS protocols to uncover shared and distinct expression patterns. The heatmap demonstrated clustering of circRNA expression patterns, clearly differentiating samples processed with Traditional and NICO APS protocols (Figure 3E). Moreover, the volcano plot shows differential circRNA expression, pinpointing specific molecules whose abundance was significantly altered between the 2 methods (Figure 3F). These findings highlight the importance of how molecular profiles are significantly impacted by the choice of extraction biopsy method, with the NICO APS protocol contributing to higher specificity in detecting differentially expressed circRNA molecules.

### circRNA-associated hub genes in WHO grade 2 meningiomas

CircRNA expression and function could differ according to WHO grade meningioma being associated with biological process linked to tumor aggressiveness. We therefore took the opportunity to explore pattern differences in WHO grade 2 meningioma relative to grade 1. To achieve this, we performed differential gene expression analysis (Figure 3G) and analyzed the results for enriched biological processes and pathways using GO and KEGG databases. The GO and KEGG enrichment analyses in the circRNA revealed that energetic and metabolic processes were downregulated in grade 2, while nuclear processes and the DNA repair response were upregulated in grade 2 (Figure 3H). These results indicate that cellular stress may be associated with tumoral aggressiveness and therapeutic resistance. To explore the co-expression relationships among circRNA modules and clinical characteristics, we performed WGCNA analysis. The dendrogram (Figure 4A) illustrates the hierarchical clustering of circRNAs into distinct modules, represented by color modules. Each module signifies a subset of circRNAs with the correlated expression patterns. The heatmap (Figure 4B) illustrates the Pearson correlation analysis employed to examine the association between these modules and specific clinical characteristics with the associated *P* value. This analysis revealed significant associations between circRNA modules and specific clinical features. Red (*R* = 0.48, *P* = .009) and salmon (*R* = 0.49, *P* = .007) modules were significantly correlated with tumor grade (grade 2), while cyan modules were positively (*R* = 0.4, *P* = .03) and blue module was negatively associated with the tumor extraction technique (Traditional vs NICO APS) (*R* = −0.55, *P* = .002). Additionally, the ANOVA comparison between each clinical feature and the 16 module colors formed with this dataset confirmed the correlation analysis (Figure S3A-E). These results suggest that circRNA expression patterns differ according to tumor grade and may also be influenced by the tumor extraction technique, suggesting that both biological and technical clinical factors are associated with distinct circRNA modules.

**Figure 4:**
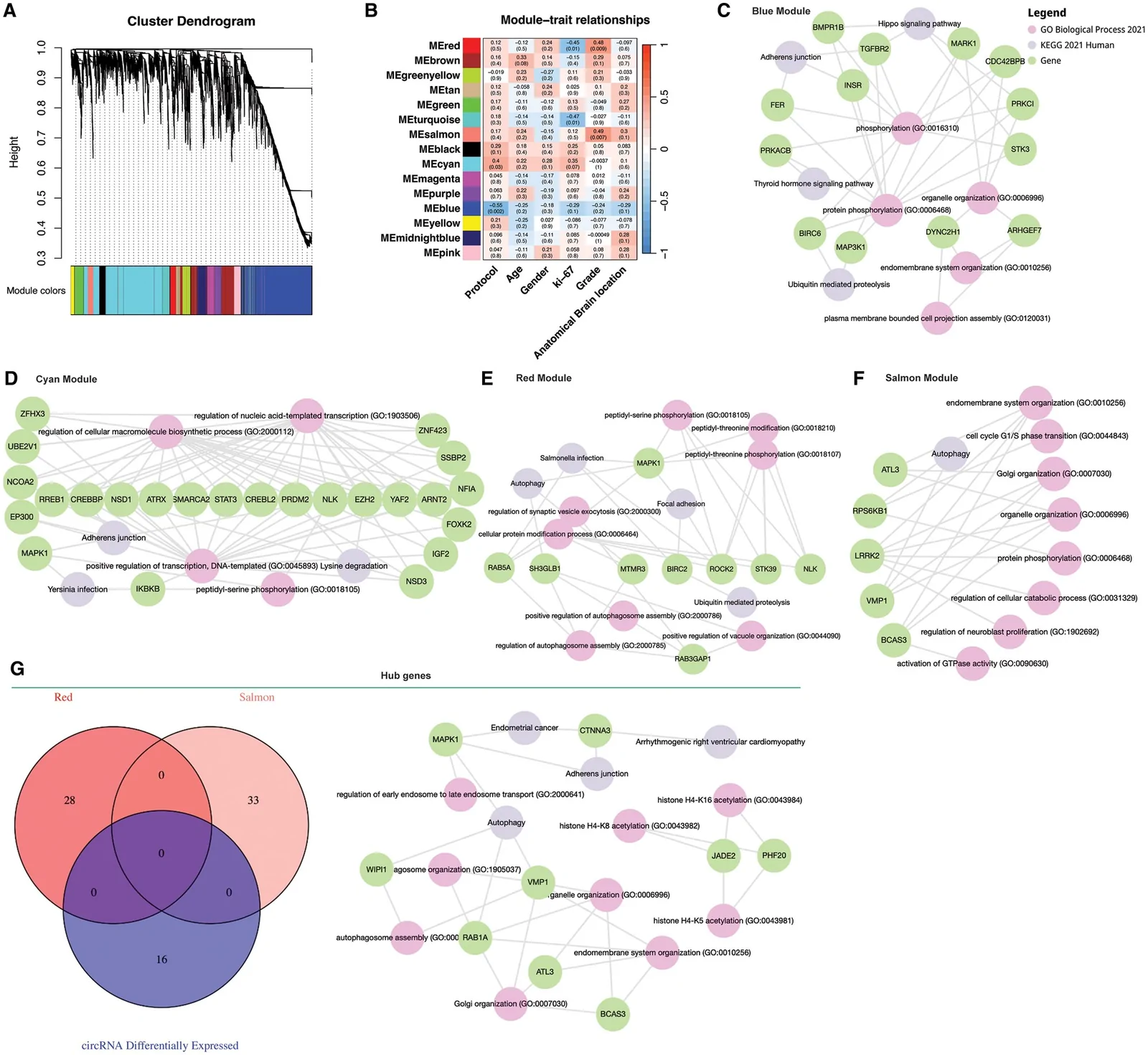
circRNA co-expression network analysis associated with clinical features. (A) The cluster dendrogram shows the 16 modules identified by the co-expressed circRNAs followed by the workflow that associated these modules with the clinical data, including age, gender, tumor WHO grade, anatomical location, and ki-67 proliferation biomarker. (B) Heatmap result of the identified module associated with clinical data. Red shading indicates a positive correlation, while blue denotes a negative correlation. Network graphical of the GO and KEGG pathways enriched (C) blue and (D) cyan module correlated with protocol; (E) red and (F) salmon module associated with WHO grade 2. (G) Venn diagram showing the number of hub genes and the enrichment network analysis in red, salmon, and differential expressed circRNAs associated with WHO grade 2.

To investigate biological functions associated with the circRNA expression patterns within modules associated with clinical features, we performed GO and KEGG pathway enrichment network analysis. Cyan module was positively correlated with NICO APS protocol enriched by transcript regulation and phosphorylation, while the blue module was negatively associated with NICO APS protocol enriched by cell organization (Figure 4C and D). We also observed that hub genes in red and salmon module correlated with WHO grade 2 are related with cellular signaling and metabolism pathways (Figure 4E-G). These findings suggest that differences in preservation protocols may influence the molecular activity observed in the samples, potentially reflecting cellular stress responses. This effect may be more pronounced in samples processed using the traditional method, which could impact the circRNA expression profile. Otherwise, patients classified as WHO grade 2 have a circRNA profile regulating genes associated with cancer cell survival.

## Discussion

In this study, we highlight the significance of tissue quality in sample acquisition, as achieved by NICO APS, as well as the application of proper normalization and batch correction strategies for circRNA. Recent neuropathological practice guidelines published by cIMPACT-NOW significantly improve prognostication. However, these guidelines leave several key questions unanswered. Chief among them is a straightforward, low-cost workflow to identify which patients should undergo extensive molecular analyses. In the United States, denial of Medicare claims for cancer related next generation sequencing (NGS) tests is over 20%, with solid tumor denials recorded at over 70%. This leaves the financial burden of testing on the patient or hospital. This is particularly problematic for specimens with grade 1 morphology, as designating a benign tumor grade will increase the probability of reimbursement denial for molecular testing.^50^ With US-based commercial laboratories charging $3000 per methylation test, this could result in significant financial risk to some patients.^50^ It will thus be important to develop neuropathological assays that decrease the pre-test probability for grade 2 to justify more extensive NGS-based assays such as methylation profiling with copy number variation (CNV). This finding aligns with other studies that have demonstrated that NGS to be cost-effective as a diagnostic modality at specific pre-test probability thresholds.^51^ While our study focuses on RNA integrity and molecular assay quality, we acknowledge that implementing cryopreservation and RNA-based analyses in routine pathology workflows presents logistical and financial challenges. Snap freezing and cryostorage require infrastructure and coordination that may not be readily available in all clinical settings. Nevertheless, circRNA assays may play a role in meningioma testing at this level, offering a promising avenue for biomarker discovery and risk stratification in selected cases. However, poor sample quality caused by tissue collection and preservation using FFPE, as well as the complexity nature of fresh-frozen tissue preservation methods limit molecular analysis.

Our study compares the traditional tissue procurement method for preserving meningioma tissue with the novel NICO Myriad APS protocol. This comparison of preservation methods demonstrates the important role of surgical specimen collection and preservation techniques in obtaining higher quality circRNA profiles. We note that the higher variation between samples and lower expression in the Traditional protocol compared to the NICO APS protocol is likely related to false-positive detections in the Traditional protocol. The bias could occur when RNA integrity affects the ribosomal RNA and RNase R digestion, causing a contamination by other types of non-circRNA, decreasing the precise detection of circRNA. It is important to consider that machine learning-based bioinformatics tools may be sensitive to RNA degradation. Degradation and the presence of short fragments resulted in increased mapping errors with subsequent false-positive transcript detection.^52^^,^^53^ For this reason, developing a gold standard of circRNA profiles are essential to improve the performance of circRNA as a biomarker. These tools are necessary to mitigate technical variability between different batch analyses, ensuring more reproducible and accurate conclusions in circRNA detection studies (Figure 5). Additionally, although our study focuses on RNA integrity and circRNA profiling, it is important to consider whether the improved preservation observed with the NICO APS system may also benefit other molecular assays, including DNA-based analyses, chromosomal arrays, and methylation profiling. While further validation is needed, the enhanced tissue integrity provided by NICO APS could potentially support multi-omic approaches from a single sample, reducing the need for separate tissue processing workflows. This would be particularly valuable in clinical settings where sample availability is limited and cost-effectiveness is essential.^54^^,^^55^

**Figure 5:**
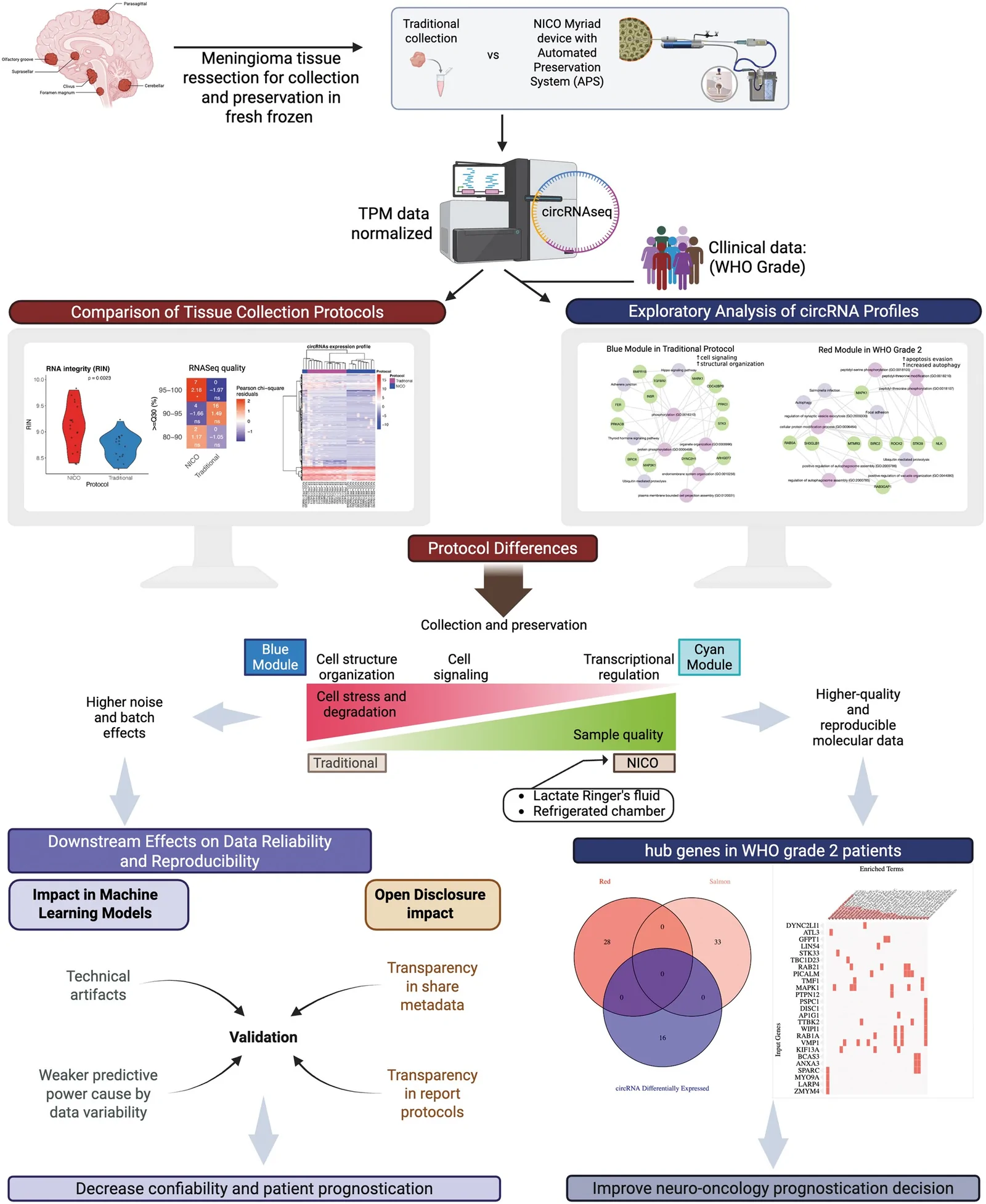
Summarizing the optimization of meningioma tissue preservation for circRNA profiling. This study demonstrates that the NICO Myriad and APS protocol provides a high-quality collection and preservation environment (Equipment image sourced from the NICO Corporation webpage: https://niconeuro.com/our-integrated-system/), significantly refining circRNA analysis in meningiomas by enhancing RNA preservation and reducing technical noise. Traditional methods introduce higher cellular stress and degradation, contributing to increased batch effects and technical noise in RNA-seq data. This variability compromises the reliability of circRNA detection, affecting the robustness of downstream analyses. In contrast, the NICO APS protocol preserves RNA quality more effectively, as demonstrated by significantly improved RNA integrity, sequencing quality. Samples collected using the traditional method exhibit a module enriched in genes related to cell structure organization, signaling disruptions, and stress responses, reflecting increased degradation. In contrast, the NICO APS protocol is linked to a module, featuring genes involved in transcriptional and epigenetic regulation, supporting the hypothesis that improved tissue preservation enhances the molecular stability of circRNA. Notably, additional circRNA modules correlate specifically with WHO grade 2 meningiomas, revealing enrichment in pathways associated with cellular metabolism and epitranscriptomic regulation, suggesting potential prognostic markers. The implications of these findings extend beyond molecular profiling. The high-quality, reproducible data obtained using the NICO APS protocol enhances machine learning models for meningioma prognosis, reducing the impact of batch effects and technical artifacts. Greater RNA stability leads to more consistent biomarker detection, ultimately improving predictive power for patient outcomes. Moreover, transparency in metadata sharing and reporting protocols strengthens data validation and facilitates the integration of circRNA-based profiling into clinical workflows. If validated in larger cohorts, this approach could redefine tumor classification, improve prognostic accuracy, and optimize neuro-oncology treatment strategies, paving the way for precision medicine applications in meningioma management. Created in https://BioRender.com.

The NICO APS provides a higher quality and more accurate foundation for downstream circRNA detection and analysis. The significant modules correlated with NICO APS protocol highlights the superiority of this protocol in correlating with clinical outcomes and biological functions, mainly related to transcript regulation associated with circRNA profile. We did not observe significant differences in circRNA profiles related to anatomical locations. Furthermore, meningiomas may be classified as grade 2 based on various distinct methodologies that could affect circRNA expression profiles. Future studies with sufficiently powered with various types of grade 2 tumors would be required to answer these questions. Nevertheless, our findings support high-quality tissue preservation improved the quality of our biomarkers, which ultimately enhances the validity of molecular assays that drive clinical decision-making.

## Supplementary Material

nlag030_Supplementary_Data

## Data Availability

The datasets generated during and/or analyzed during the current study are available in the GEO repository #GSE298049. Full code and packages used for R analysis can be found on GitHub: https://github.com/GlauciaMMFernandes/circRNA-in-meningioma-profile.
