# Supplementary material for: NICO Myriad Automated Preservation System improves meningioma sample quality for molecular pathology assays
Source: J Neuropathol Exp Neurol. 2026 Jul 20;85(11):1278–90. doi: 10.1093/jnen/nlag030 (PMC13678740; doi:10.1093/jnen/nlag030)
Supplement: Supplementary file 1 [file nlag030_supplementary_data.zip › MS26-035R2 Otero Supplementary Figure 3.docx]

**Supplementary Figure 3. Biological process enriched in significative module.** Greenyellow module (A) network analysis and (B) barchart of GO and KEEG enrichment ranked according to their p-values (p < 0.05), (C) gene significance and (D) barplot comparison between protocols correlated to ki-67 biomarker. Magenta module (E) network analysis and (F) GO and KEEG enrichment ranked according to their p-values (p < 0.05), (G) gene significance and (H) barplot comparison between protocols correlated to ki-67 biomarker. Test t comparing the significance of the modules colors between (C) advance age <50 years, (D) WHO grade, (E) ki-67 proliferation biomarker, (F) Traditional or NICO APS protocol, (G) gender, and (H) anatomical location. Initial comparisons were screened by ANOVA and significant effects are indicated by *p < 0.05; **p < 0.01; ***p < 0.001; **** p < 0.0001.
