# Supplementary material for: NICO Myriad Automated Preservation System improves meningioma sample quality for molecular pathology assays
Source: J Neuropathol Exp Neurol. 2026 Jul 20;85(11):1278–90. doi: 10.1093/jnen/nlag030 (PMC13678740; doi:10.1093/jnen/nlag030)
Supplement: Supplementary file 1 [file nlag030_supplementary_data.zip › MS26-035R2 Otero Supplementary Figure 1.docx]

**Supplementary Figure 1. Mitigate batch effects and normalization.** (A) RLE plot of raw (top), after batch mitigation (middle) and normalization (bottom). (B) Distribution of PCA analysis raw (left), after batch mitigation (middle) and normalization (right) showing the improvement in the samples distribution after applying Combat and quantile normalization to mitigate technical biases.
