# Supplementary material for: NICO Myriad Automated Preservation System improves meningioma sample quality for molecular pathology assays
Source: J Neuropathol Exp Neurol. 2026 Jul 20;85(11):1278–90. doi: 10.1093/jnen/nlag030 (PMC13678740; doi:10.1093/jnen/nlag030)
Supplement: Supplementary file 1 [file nlag030_supplementary_data.zip › MS26-035R2 Otero Supplementary Figure 2.docx]

**Supplementary Figure 2. Clustering.** (A) PCA plot distribution of the hierarchical clustering showing the inter-cluster centroids Euclidian distance and the intra-cluster mean distance. (B) Boxplot showing the silhouette analysis between the hierarchical clustering. T test were used for specific groupings and significant effects are indicated by ns= non-significant; *p < 0.05; **p < 0.01; ***p < 0.001; **** p < 0.0001.
